# It Keeps the Good Boy Healthy from Nose to Tail: Understanding Pet Food Attribute Preferences of US Consumers

**DOI:** 10.3390/ani11113301

**Published:** 2021-11-19

**Authors:** Meike Rombach, David L. Dean

**Affiliations:** 1 Department of Land Management and Systems, Lincoln University, Lincoln 7647, New Zealand; 2 Department of Agribusiness and Markets, Lincoln University, Lincoln 7647, New Zealand; david.dean@lincoln.ac.nz

**Keywords:** pet food, pet food involvement, pet owner, product attributes

## Abstract

**Simple Summary:**

Understanding the importance consumers place on pet food product attributes is relevant for marketing managers in food and specialized pet supplies retailers, as this information allows them to identify how to modify or develop products to match the needs and wants of different consumer groups. The study proposes a model that investigates the importance pet owners place on convenience, natural ingredients, and claims on the packaging (such as value and healthiness) as product attributes.

**Abstract:**

The study provides insights for marketing managers in specialized pet supplies retailers, as well as for vets and animal welfare organizations. This study proposes a model that investigates the importance pet owners place on convenience, natural ingredients, and value and health claims as product attributes. For this purpose, an online survey with a sample size of 206 pet-owning US residents was conducted. Partial least squares structural equation modelling shows that pet food purchase involvement positively impacts subjective and objective knowledge about pet food. Subjective knowledge appears to be the strongest factor impacting the importance consumers place on all three attributes. This is followed by objective knowledge. Socio-demographic factors such as gender, age, income, and education appear to have a limited impact as predictors for the importance consumers place on the product attributes.

## 1. Introduction

The US can be described as a nation of animal enthusiasts, with the highest proportion of households with pets in the world [1]. Pets have an important role in US society [2,3], given that 85 million people own at least one pet and the trend of “pet parenting” has been vastly increasing in recent years [4,5,6]. Along with this trend and the rise of animal welfare concerns [7,8], feline and canine owners have become more mindful in their choices for medical care, pet supplies, other pet services such as grooming and boarding, and pet food [9,10,11,12,13,14].

In 2020, pet food and treats accounted for sales of $44.1 billion in the US pet food market [15]. Pet owners had expenses ranging from $254 to $287 per annum for basic pet food [12] and may have paid an extra $120 to $160 for treats and vitamins [6]. Different pet owners, however, consider different types of pet food, with varying product attributes to fulfil the nutritional requirements of their pet [16,17]. The most important product attributes appear to be price, perceived ingredient safety, and perceived quality and nutritional value [17,18,19,20,21].

Most commercial pet foods are formulated based on the nutritional composition of ingredients available in public databases [17]. Ingredient composition and pet food quality are key for many pet owners when choosing between raw, wet, or dry food [22], and they perceive certain ingredients as undesirable or unsafe [23]. Ingredients such as wheat and corn may be perceived as low quality or fillers by some pet owners [19], although these claims are not scientifically based [24]. However, they may still appreciate dry pet foods with cereals due to affordability and convenience [25]. Other important product attributes pet food owners search for when inspecting suitable pet food items are often related to the production and processing of the product. Specifically, natural, wholesome, organic, or cruelty-free pet food are gaining popularity [26,27]. Vinassa et al. (2020) highlighted that social and cultural factors such as the eating habits of pet owners influence the decision-making processes when buying pet food and feeding practices [19]. Other key factors that determine the pet food preferences of US consumers are widely unexplored. Therefore, the present study focuses on this literature gap and explores which key factors determine the importance US consumers place on pet food attributes.

### 1.1. Key Factors Determining the Importance US Consumers Place on Pet Food Attributes

A US consumer’s pet food purchase involvement, their objective and subjective knowledge, as well as their sociodemographic backgrounds are likely to be key factors determining the importance they place on pet food attributes. In the remainder of this section these factors are explained in more detail, as these serve to build the conceptual framework.

### 1.2. Pet Food Purchase Involvement

Pet food purchase involvement is defined as the extent to which pet owners devote interest and effort into purchasing pet food [28]. According to Montandon et al. (2017), involvement is high when a product is highly valued, and considerable research is required prior to purchasing [29]. Often, more expensive or riskier purchases are considered high involvement. Alternatively, involvement is considered low [30,31] when a product is bought habitually, requiring little or no previous research. These low involvement purchases often represent minor expenses and risk [32].

Purchase intent, use, value, pleasure, and integrity associated with a product determine how the product is evaluated by pet owners. The evaluation depends on the pet owner’s extent of involvement [33]. Pet owners showing high involvement tend to have a greater interest in product information. They are likely to compare and contrast product attributes and features and hold favourable or unfavourable views towards them [34]. Regardless of whether the involvement is low or high, consumers experience positive or negative emotions about a purchase and this impacts their satisfaction or dissatisfaction with the transaction. Positive emotions exert a higher influence on satisfaction in low involvement products than in high involvement products [34].

Pet food purchase involvement has been studied by Dotson and Hyatt (2008) [35]. Their study was dedicated to understanding canine-human relationships. It was found that if pet owners view their pets as an extension of themselves, they spend an increased amount of time, effort and money making sure the lifestyle of their pets resembles their own. This includes providing snacks, treats, home-cooked meals, and branded foods [35]. This has been confirmed by other studies investigating lifestyle, dietary, and human-animal relationships [36,37,38,39,40]. Further evidence stems from veterinarian research. Veterinarian studies have found that pet owners actively seek specific product attributes when purchasing pet food and consider their vet and the Internet as essential information sources when it comes to pet food purchases [19,41]. These findings have been confirmed by Park and Um (2021) and Kwak and Cha (2021) in their research on pet food purchases and consumer behaviour in times of COVID-19 [25,42]. Park and Um’s research (2021) adds value to the body of literature on pet food [25], as the study presents consumer clusters based on varying degrees of interest and involvement in pet food purchasing. These include a cluster of pet food buyers with little interest and involvement, a cluster of pet food information- and price-seeking consumers, a pet food convenience-seeking cluster, and pet food higher-involvement cluster [25].

### 1.3. Objective and Subjective Consumer Knowledge

A pet owner’s knowledge can influence their attitudes toward pet food and therefore the importance they place on pet food attributes. Consumer knowledge is closely related to perception. Depending on their knowledge and perception, consumers may evaluate pet food product attributes either negatively or positively [43]. When consumers have a high level of involvement with a certain product that is of personal interest, product knowledge increases [43]. Consumers are often concerned about buying unfamiliar products when they lack sufficient knowledge to anticipate negative impacts [43]. Similarly, they may view familiar products favourably and develop loyalty towards the product. Consumer knowledge is commonly distinguished between objective and subjective knowledge [44]. Subjective knowledge is also known as perceived knowledge because it describes the consumer’s perception of their knowledge, whereas objective knowledge refers to what they actually know [45,46,47].

Objective knowledge is stored in the consumers’ long-term memory and is usually assessed through testing [48,49]. In contrast, subjective knowledge is based on direct experience by consumers and their interpretation of these experiences [43]. In research, subjective knowledge is commonly assessed through self-reporting [50,51]. Self-report relies on an individual’s own report of behaviours, attitudes, or as in case of the present study, what participants believe they know [50].

The body of literature on objective and subjective knowledge is not conclusive. While some studies suggest both types of knowledge are interconnected, other studies have shown that they can be different [43,50]. The latter branch of literature is supported by the Dunning-Kruger effect which suggests that consumers may think they have sufficient knowledge while knowing very little. In addition, increased objective knowledge may lead consumers to re-evaluate and sometimes downgrade their subjective knowledge [43].

In a pet food context, Surie (2014) addresses the discrepancy between subjective and objective knowledge [52]. While pet food owners self-reported high pet food knowledge of different types of pet food and their healthiness, objective knowledge does not match these claims [52]. Similarly, several other studies found that knowledge about pet food and pet food attributes among pet owners is varied [53]. While some owners have good knowledge, others need to improve their feeding and food storage practices and educate themselves on the health, safety, and nutrition of certain pet food types and their attributes [19,54,55].

For instance, Morelli et al. (2021) found that pet owners should be educated in proper food storage management and receive feeding instructions from veterinarians [17]. Exposure to direct light and exposure to high temperatures were emphasised as mistakes in the storage of pet food, indicating a pet owner’s lack of knowledge [17]. Thomas and Feng (2021) researched pet food knowledge in a food safety context [56]. Their study found that there is consumer confusion about the safety and healthiness of dry and raw food. Some pet owners were unaware of pet food recalls or outbreaks associated with foodborne pathogens. In particular, dry foods and treats were not identified as potential sources of foodborne illnesses [56]. Similar findings have been emphasized in studies on pet food and mycotoxins [57,58].

### 1.4. Socio-Demographics

Several studies have reported socio-demographic backgrounds of people buying pet food in the US, but the body of literature is rather inconclusive. Some studies indicated pet food purchase is associated with gender, age, income, and education [41]. Being female, primary caretaker of the pet, having a high income, and high level of education have been cited as determinants of pet acquisition [17,41]. Other studies suggest that socio-demographics do not have much impact. These studies emphasize owner dietary preferences, attitudes, and lifestyle as key-factors impacting pet supply purchases including food [14,40,59,60,61,62]. If drivers of pet food purchasing behaviour is more complex than previously assumed, this may explain the conflicting results reported by research relying solely on socio-demographic predictors. This suggests that future models that combine socio-demographics with consumer attitudes and preferences, such as pet food purchase involvement and knowledge, may be more fruitful.

### 1.5. Importance Pet Owners Dedicate to Intrinsic and Extrinsic Pet Food Attributes

When pet owners purchase food for their animal companions, they commonly evaluate pet food products based on a combination of intrinsic and extrinsic product attributes. Intrinsic product attributes specify the physical aspects of the product, for instance chemical composition, aroma, and nutritional properties [63,64]. Extrinsic attributes are related to the product itself, but physically are not a part of it. Brand, price, and product claims related to sustainability, animal welfare, and production practices are examples of extrinsic attributes which are relevant for pet owners buying pet food [64,65].

Vinassa et al. (2020) studied the perceptions dog and cat owners had of different product attributes to understand what consumers perceive as pet food quality [19]. The study included product claims such as cruelty-free, organic, health benefits, wholesomeness, perceived ingredient safety, nutritional information, price, brand, information, smell, and the food’s impact on the pet’s stool appearance. They found that while older consumers sought value for money, younger consumers paid most attention to the stool quality, the percentage of protein in the feed, and the use of recyclable packaging [19].

The importance of natural ingredients and value for money, which in pet food is often the perceived health benefits relative to cost, have been highlighted in earlier studies [53,54]. Vinasse et al. (2020) found that overall, “natural” ingredients were the most important attribute when determining pet food quality [19]. Pet food appearance, smell, a higher cost, and information about protein content, the presence of fresh meat, and being free of unwanted fillers were of interest for buyers of wet pet food [19]. Other studies highlight the importance of dry food such as kibble, as this type of food is cheap and convenient [56,66,67,68]. Surie (2014) found that most consumers with high levels of subjective pet food knowledge mostly fed their pets raw food or other food forms with natural ingredients, but some fed their pets dry food [52]. Pet owners who fed mostly raw food assumed that dry food contains only low-cost product fillers such as wheat and grains [52].

### 1.6. Conceptual Framework

**Figure 1:**
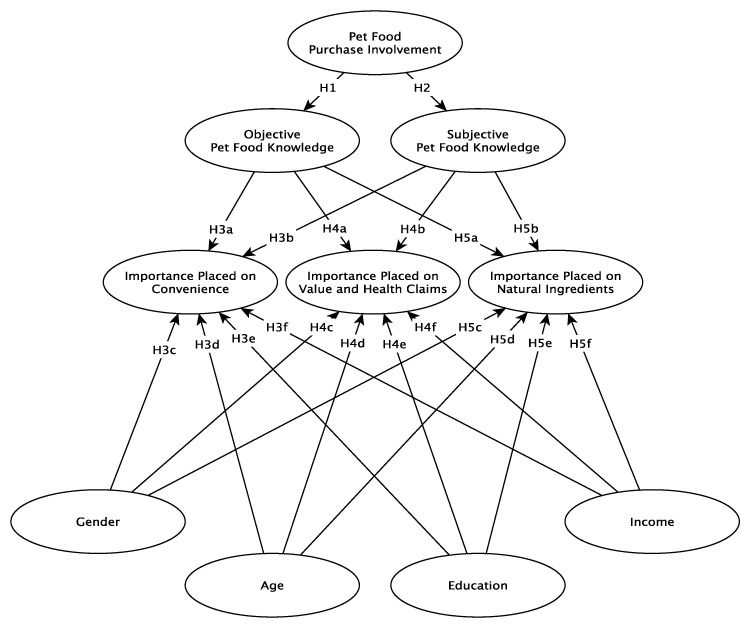
Conceptual Framework.

A conceptual framework based on the literature is proposed. It is suggested that the importance that US pet owners place on pet food attributes such as convenience, natural ingredients, and value and health claims are likely to be influenced by socio-demographic backgrounds of consumers, their involvement in pet food purchases, and their objective and subjective knowledge about pet food (see Figure 1). While the proposed conceptual framework attempts to incorporate all of the relevant research findings into a comprehensive set of relationships, the socio-demographic constructs and relationships do not have the same depth of literature support and are somewhat exploratory. The following hypotheses are proposed:

**Hypothesis** **1** **(H1).** *Pet food purchase involvement is likely to positively impact objective knowledge*.

**Hypothesis** **2** **(H2).** *Pet food purchase involvement is likely to positively impact subjective knowledge*.

**Hypothesis** **3** **(H3).** *The importance that consumers place on convenience as a product attribute is likely to be positively impacted by (a) objective knowledge, (b) subjective knowledge, (c) gender, (d) age, (e) education, and (f) income.*

**Hypothesis** **4** **(H4).** *The importance that consumers place on value and health claims as a product attribute is likely to be positively impacted by (a) objective knowledge, (b) subjective knowledge, (c) gender, (d) age, (e) education, and (f) income.*

**Hypothesis** **5** **(H5).** *The importance that consumers place on natural ingredients as a product attribute is likely to be positively impacted by (a) objective knowledge, (b) subjective knowledge, (c) gender, (d) age, (e) education, and (f) income.*

## 2. Material and Methods

### 2.1. Survey Instrument and Data Collection

An online survey in Qualtrics was used to gather information in July 2021 from a sample of U.S. residents targeted for pet ownership. The survey was distributed via Amazon Mechanical Turk, a crowdsourcing platform, and designed to collect socio-demographic information as well as respondents’ knowledge about pet food, their feeding practices, and preferences for pet food types and product attributes. Respondents had to be US residents and at least 18 years old to participate. The data collection resulted in 206 completed responses (156 males and 50 female respondents), which were considered appropriate for this research, given that all respondents indicated they owned at least one cat or dog. The sample of US citizens is appropriate for exploring key factors impacting attributes pet owners deem important via partial least square structural equation modelling (PLS-SEM), because a standard method in PLS-SEM to determine the minimum sample size has been employed [69]. The “10-times rule” method following Hair et al. (2011) [70], which builds on the assumption that the sample size should be greater than 10 times the maximum number of inner or outer model links pointing at any latent variable in the model [70]. In this research, the maximum number of links was 9 (3 outer and 6 inner links), so a minimum sample of 90 was more than satisfied.

### 2.2. Construct Measurement

While many of the constructs have been discussed in the literature, validated scales to adopt for the current research were only partly available. Thus, measurement items were developed from the relevant concepts proposed in the literature. Pet food purchase involvement (3 items) was measured using 6-point non-specific frequency scales (1 = never to 7 = always) and subjective knowledge (3 items) was measured using 7-point Likert scales (1 = strongly disagree to 7 = strongly agree). Objective knowledge (4 items) was measured by asking respondents to rate the difference between 2 food types on 7-point similarity scales (1 = very dissimilar to 7 = very similar) and scoring their responses.

The 9 pet food product attributes importance items were measured using 7-point importance scales (1 = extremely unimportant to 7 = extremely important). Prior to the model measurement and structure analyses, the 9 pet perceptions and engagement items were subjected to a factor analysis using principle components extraction (Eigenvalues > 1) and varimax rotation in SPSS, resulting in three factors, which were named importance placed on convenience of pet food, importance placed on value and health claims of pet food, and importance placed on natural ingredients.

### 2.3. Data Analysis

Descriptive statistics and PLS-SEM were used to analyse the data. Partial least square structural equation modelling is a standard method in various social science disciplines to analyse complex interrelationships between observed and latent variables [71,72]. According to Hair et al. (2019), it is a causal-predictive approach that allows for prediction in estimating statistical models [73]. The approach overcomes the dichotomy between explanation and prediction, which is essential in the development of best practice recommendations and managerial implications. PLS-SEM is particularly appropriate due to a number of characteristics of the current research and data, namely the relatively small sample size and the exploratory nature of the research in a relatively unexplored field. Additionally, the non-normal distribution of the data and combination of Likert scales, difference scales, and single item demographic data preclude the use of maximum likelihood SEM modelling [72].

Partial least square structural equation modelling is a combination of path analysis and principal component and regression analysis, and follows a two-step approach [74]. The first step was dedicated to the outer model and consisted of checking reliability and validity via measurement model functions. Indicator loadings of greater than 0.4 verified indicator reliability. In terms of internal consistency of the model, the average variance extracted (AVE > 0.5), construct reliability (Cronbach’s Alpha > 0.6), and composite reliability (CR > 0.6) were used to test the convergence criterion [75,76]. Convergent validity explains the extent a construct converges to explain the variance of its items [73]. Following Hair et al. (2019b), composite reliability values between 0.60 and 0.70 are considered “acceptable in exploratory research,” and values between 0.70 and 0.90 range from satisfactory to good. A value higher than 0.95 suggests that the item is redundant; negatively impacting construct validity [74].

In order to test the constructs within the model, the Fornell-Larcker criterion and cross-loadings determining discriminant validity need to be evaluated [75]. When testing discriminant validity by checking cross-loading, all items should have a higher correlation with their assigned factor than with other factors. The Fornell-Larcker criterion is fulfilled if the square root of each construct’s AVE is greater than the correlation with other constructs [75,76,77]. Following Henseler et al. (2015), the heterotrait-monotrait ratio of correlations criterion (HTMT) with a threshold value of 0.9 was used to confirm discriminant validity [75]. Finally, multicollinearity was checked with the Variance Inflation Factor (VIF), which is recommended to be under 5 [75].

The second step focused on the inner model to determine the structural fit of the model [76]. To evaluate the model quality, the model fit is reported and the explanatory power is evaluated. Hair et al. (2017) caution the interpretability of model fit indices in SEM-PLS [76], but convention suggests that goodness of fit (GoF) and normed fit index (NFI) are reported and both GoF and NFI scores vary from 0 to 1, where closer to 1 is considered a better fit. Additionally, the standardised root mean square residual (SRMR) is reported where a value of more than 0.10 is problematic and less than 0.08 is considered acceptable. The explanatory power of the model is evaluated by the individual and average variance explained (R^2^) of the dependent variables, with values of 0.75, 0.5, and 0.25 considered substantial, moderate, and weak [74]. Predictive validity is assessed using the Stone Geisser criterion (Q^2^), which, if larger than zero for an endogenous latent variable, indicates that the model has adequate predictive relevance for the construct [74]. Further, Q^2^ scores larger than 0.25 and 0.5 indicate medium and large predictive accuracy respectively. The software packages SPSS and SmartPLS were used to examine the research model and test the proposed hypotheses.

## 3. Results

Table 1 shows the sample description statistics with a median respondent aged 25–34, with a bachelor degree and an annual pre-tax income between $25 k and $50 k per year.

The assessment of the outer model included the reliability and the convergent and discriminant validity of the scales used to measure the constructs present in the proposed conceptual framework. Table 2 shows the item-factor loadings were all above the minimum 0.4, indicating suitable items in each scale. With the exception of the Cronbach Alpha score of subjective knowledge (0.587) and importance placed on value and health claims of pet food (0.574), all the other Cronbach Alpha scores and all the composite reliability scores were above 0.6, confirming reliability. All the AVE scores were above 0.5, indicating convergent validity.

Discriminant validity was tested using the Fornell-Larker Criterion and Heterotrait-Monotrait ratios. Table 3 shows that the requirements of the Fornell-Larker criterion were satisfied. Further, the HTMT ratios confirmed acceptable discriminant validity, except for higher than recommended HTMT ratio between subjective pet food knowledge and natural ingredients importance (0.970). While concerning, discriminant validity was largely confirmed. The constructs that were not confirmed were considered to be conceptually distinct, so discriminant validity was deemed satisfactory. Finally, multi-collinearity was not seen as a problem as the highest VIF score was 1.899; well below the recommended maximum of 5.

The structure of the conceptual framework was tested resulting in a goodness of fit of 0.428, a normed fit index of 0.665, and a standardised root mean square residual of 0.082, indicative of an adequate overall model fit. Tests for the explanatory and predictive power of the conceptual model resulted in R^2^/Q^2^ values of 0.424/0.232 for convenience importance, 0.404/0.215 for natural ingredients importance, 0.296/0.219 for objective pet food knowledge, 0.238/0.117 for subjective pet food knowledge, and 0.152/0.049 for value and health claims importance. This confirms that the explanatory power of the model is weak to moderate and the predictive accuracy is considered weak to medium. Thus, the structure of the model is confirmed to be fit for hypothesis testing.

**Figure 2:**
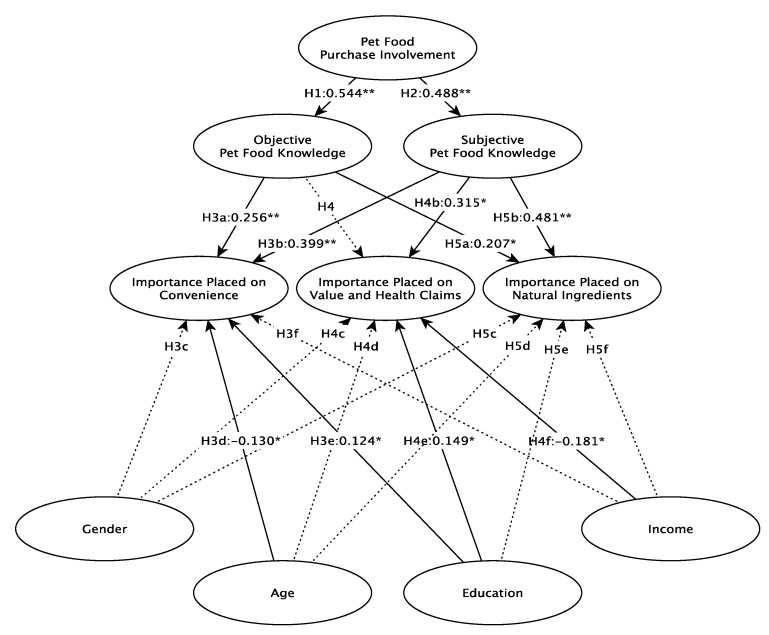
Conceptual Model Results, * = *p* < 0.05, ** *p* < 0.01, dotted line = not significant.

Table 4 and Figure 2 show the results of the hypothesis testing. Pet food purchase involvement contributes to both objective and subjective knowledge, supporting hypotheses H1 and H2. Importance of convenience is influenced by subjective and objective knowledge, and is more important for younger and more educated pet food buyers, supporting H3a, H3b, H3d, and H3e. Importance of value and health claims is influenced by subjective pet food knowledge, and is more important for more educated and lower income pet food buyers, supporting H4b, H4e, and H4f. Finally, importance of natural ingredients was influenced by both subjective and objective knowledge but none of the demographic characteristics, supporting H5a and H5b.

## 4. Discussion

This study seeks to understand key factors determining the importance that US pet owners place on intrinsic and extrinsic pet food attributes. Overall, the proposed model was found to have an adequate fit and explanatory power. Results emphasize the importance of pet food purchase involvement, as well as pet owners’ objective and subjective knowledge about pet food as factors impacting the importance US pet owners place on convenience, value and health claims, and natural ingredients as product attributes. Only some socio-demographic factors were found to have an impact.

The model confirms previous findings that purchase involvement has an impact on knowledge. A high level of involvement implies some experience with pet food, and this contributes to both objective and subjective knowledge. Specifically, a pet owner’s direct and indirect experiences with pet food are positively associated with the level of product information stored in their memory and their self-assessment of that knowledge [47,78].

Involvement may come from searching for information prior to and during purchase decisions [25], including personal experience, exposure to other owner experiences, product advertisement, retail store displays [34], or advice from the vet [17].

It is noteworthy that both objective and subjective knowledge are the strongest predictors for the importance that US pet owners place on convenience and natural ingredients as product attributes. These findings corroborate recent studies on pet food attributes by Park (2021) and Vinassa et al. (2021) which outline the importance of both attributes [17,19]. Subjective knowledge has an impact on value and health claims, but objective knowledge did not show any significant impact. Given that objective knowledge is fact-based, retained in long-term memory, and implies theoretical or practical understanding of a subject [54], in this case pet food, pet owners with objective knowledge may not find claims of any kind important. Perhaps claims are viewed as reasoned extrapolations of knowledge, which are not necessarily fact-based. Claims related to the healthiness of raw food diets for pets, or the unhealthiness of dry food items are hard to evaluate for consumers, as these topics have long been debated in the pet food industry and among scientists without reaching a consensus [19,79].

Value for money claims can be difficult to evaluate because consumers may be promotion- or prevention-focused when it comes to value for money claims [80]. Both stances are relevant to the strategies pet owners use to reach their desired outcome. Pet owners may either strive for results that match their desired outcome (promotion focus) or avoid results that do not match their desired outcome (prevention focus) [80]. If a consumer’s desired outcome is “getting enough value for money by choosing the right pet food product”, then certain retail practices may not satisfy them. For instance, a reduction of portion size without a commensurate reduction in price may disappoint both types of consumers [81,82].

Findings concerning the socio-demographic backgrounds of pet owners and their impact on the importance dedicated to convenience, value and health claims, and natural ingredients as product attributes are only partially confirmed in recent studies. This can be in part attributed to the diversity in findings and inconclusiveness of the body of literature on pet acquisition and pet food. Gender was not found to have any significant impact on any of the attributes and age only showed a negative impact on convenience as a product attribute. Perhaps elderly pet owners may not be that interested in convenience because pet food per se is a relatively convenient product, coming in pouches, bags, or cans that are ready to be served without time-consuming preparation or processing requirements. Following Peura Kapanen et al. (2017) [83], elderly people have reservations about and negative associations with convenience products, as they perceive convenience and quick solutions as negative [83]. Moreover, other studies emphasize that some elderly people face problems with opening the small tight lids, reading the small print on packaging, and with spillage during opening [84,85].

Education positively impacts the importance that pet owners place on convenience, as well as on value and health claims as product attributes. Education often determines access to certain lifestyles and lifestyle choices [86]. In this lifestyle context, convenience, as well as value and health claims, are likely to be important to pet owners who view their pets as an extension of self. These owners do not shy away from effort, time, and money to spoil their pets with goods and opportunities that reflect their own lifestyle [35,40]. Convenience, value for money and health are aspects that are relevant in many lifestyles. The findings related to income suggest a negative relationship between income and value and health claims, which suggest that at higher levels of income, pet food value for money is less important.

## 5. Future Research and Limitation

The respondents in the present study were recruited using Amazon-Mechanical Turk (Mturk), a widely used crowd-sourcing platform operating since 2005. The crowd-sourcing platform attracted early criticism for its low pricing, but numerous researchers have now used it for their data collection [87]. The research on crowd-sourcing platforms conducted in the last 15 years showed that this form of data collection is on par with more traditional forms of survey method. However, it needs to be acknowledged that samples from Mturk are not equal in their representativeness of the US-population, compared to representative national probability samples and opt-panels [87]. Yet, a sample of Mturk workers tends to be more representative of the US population than college samples or in-person or online convenience samples [87]. Besides these shortcomings, the present study addresses a recent issue and corresponds to a topic area in literature that is not as widely researched. Therefore, the study in its current form still adds value to the existing body of academic literature, as well as to participants in the pet industry.

Future research may address pet food attribute preferences following Vinassa et al. (2020) [19]. This is a rather unexplored area and would allow pet food producers, processors, and marketers to develop and adjust pet food products and match them with consumer needs. Perhaps a combination of best-worst approach with a latent class analysis on pet food attributes would be suitable. Such an approach would uncover the trade-offs consumers make when choosing products and classify consumer groups by their preferences.

Additionally, the product attribute importance items were factor analyzed, resulting in three factors: importance placed on convenience of pet food, importance placed on value and health claims of pet food, and importance placed on natural ingredients. With such data reduction techniques, it is important to confirm these results in other samples and contexts so this would be a valuable research avenue.

Further research could be framed in an animal welfare context and focus on consumers’ willingness to pay for cruelty free products and claims related to production practices and sustainability. Species appropriate husbandry and sustainable food production are gaining increased consumer interest [19]. Following Park (2021) the concept of pet food involvement could be more intensively studied [25], and a national representative study of US-pet food buyers may help to shed light on the ever-present discussion on socio-demographic information and its importance in predicting pet food buying behavior.

## 6. Conclusions and Managerial Implication

The present study widened the understanding of pet food consumer behavior in the US, by analyzing the importance that US pet owners place on intrinsic and extrinsic pet food attributes. Results show that pet food purchase involvement positively affects subjective and objective knowledge about pet food. Subjective knowledge appears to be the strongest factor influencing the importance consumers place on convenience, natural ingredients, and value and health claims as product attributes. This is followed by objective knowledge. Socio-demographic factors such as gender, age, income, and education appear to have a limited impact as predictors for the importance consumers place on the product attributes. Findings of the study are of relevance to many participants in the pet industry, particularly veterinarians, animal welfare organizations, and marketing managers in specialized pet food stores or pet supplies retailers. Veterinarians and animal welfare organizations could be investing in awareness campaigns and best practice advice related to healthy feeding strategies and help clarify what is fact or fiction when it comes to choices related to raw or dry pet food. This may help to avoid undesirable feeding practices and improve animal health and wellbeing.

Marketing managers may want to consider the findings related to pet food purchase involvement, convenience, and health and value claims as they are helpful to provide offerings to different target groups. Strategies for pet food marketers should not only address ideals, aspirations, and benefits for the pet, but also the needs of the owner. For the elderly, this may be accomplished through product design, adjusting small font sizes for essential product information, and making sure the product is easy to open.

All over, the present study contributes to the existing body of literature by complementing the veterinarian and food science perspective on pet food attributes through a marketing lens. It further adds to the various consumer studies on dog-ownership, pet supplies, and animal welfare, as species appropriate feeding is essential to animal health and wellbeing.

## Figures and Tables

**Table 1:** Demographic information of study participants who completed the pet food survey.

	Freq	%	Median	StDev
Age				
Under 21	1	0.5		
21–24	9	4.4		
25–34	130	63.1	✓	0.907
35–44	43	20.9		
45–54	14	6.8		
55–64	7	3.4		
65+	2	1		
Total	206	100		
Education				
Did not finish high school	1	0.5		
Finished high school	20	9.7		
Attended University	23	11.2		
Bachelor Degree	129	62.6	✓	0.826
Postgraduate Degree	33	16		
Total	206	100		
Household Annual Income				
$0 to $24,999	39	18.9		
$25,000 to $49,999	82	39.8	✓	1.010
$50,000 to $74,999	58	28.2		
$75,000 to $99,999	20	9.7		
$100,000 or higher	7	3.4		
Total	206	100		

Note: Tick mark indicates median value.

**Table 2:** Assessment of the outer model: scale loadings, reliabilities, and convergent validity.

Scales and Items	Factor Loadings	Cronbach’s Alpha	Composite Reliability	Average Variance Extracted
Pet Food Purchase Involvement		0.879	0.925	0.806
In your household, do you typically decide which pet food gets purchased?	0.922			
In your household, do you typically purchase the pet food?	0.920			
In your household, do you typically serve the pet food?	0.849			
Objective Knowledge (ability to discern differences between food types)		0.890	0.924	0.752
Kibble and Freeze Dried	0.861			
Frozen Raw and Human Food	0.871			
Human Food and Fresh Meat Chunk	0.843			
Wet Can and Human Food	0.894			
Subjective Knowledge		0.587	0.779	0.541
I know a lot about the nutritional value of pet food	0.732			
I am confident in my knowledge about pet food	0.809			
I know that I am feeding my pet food that is best for its health and wellbeing	0.659			
Importance Placed on Convenience of Pet Food		0.683	0.823	0.608
Importance of convenient packaging	0.799			
Importance of easy to serve	0.730			
Importance of portion size	0.808			
Importance Placed on Value and Health Claims of Pet Food		0.574	0.775	0.536
Importance of price	0.721			
Importance of health benefit claims	0.814			
Importance of complete and balanced nutrition claims	0.653			
Importance Placed on Natural Ingredients		0.654	0.812	0.591
Importance of country of origin	0.834			
Importance of all natural	0.731			
Importance of named meat sources	0.737			

**Table 3:** Fornell-Larcker and Heterotrait-Monotrait Discriminant Validity Test Results.

Fornell-Larcker Criterion	Convenience Importance	Natural Ingredients Importance	Objective Pet Food Knowledge	Pet Food Purchase Involvement	Subjective Pet Food Knowledge	Value and Health Claims Importance
Convenience Importance	0.780					
Natural Ingredients Importance	0.492	0.769				
Objective Pet Food Knowledge	0.549	0.527	0.867			
Pet Food Purchase Involvement	0.325	0.310	0.544	0.898		
Subjective Pet Food Knowledge	0.586	0.614	0.681	0.488	0.736	
Value and Health Claims Importance	0.344	0.371	0.247	0.038	0.324	0.732
Heterotrait-Monotrait Ratio						
Natural Ingredients Importance	0.724					
Objective Pet Food Knowledge	0.673	0.673				
Pet Food Purchase Involvement	0.404	0.395	0.611			
Subjective Pet Food Knowledge	0.890	0.970	0.893	0.629		
Value and Health Claims Importance	0.592	0.613	0.327	0.094	0.548	

**Table 4:** Path Coefficients/Hypothesis Testing Results.

Hypothesised Relationship	Coefficient	T Stat	*p*-Value
H1: Pet Food Purchase Involvement -> Objective Pet Food Knowledge	**0.544**	12.001	0.000
H2: Pet Food Purchase Involvement -> Subjective Pet Food Knowledge	**0.488**	10.264	0.000
H3a: Objective Pet Food Knowledge -> Convenience Importance	**0.256**	2.606	0.009
H3b: Subjective Pet Food Knowledge -> Convenience Importance	**0.399**	4.620	0.000
H3c: Gender -> Convenience Importance	−0.035	0.603	0.547
H3d: Age -> Convenience Importance	**−0.130**	2.229	0.026
H3e: Education -> Convenience Importance	**0.124**	2.148	0.032
H3f: Income -> Convenience Importance	−0.074	1.258	0.208
H4a: Objective Pet Food Knowledge -> Value and Health Claims Importance	0.044	0.370	0.711
H4b: Subjective Pet Food Knowledge -> Value and Health Claims Importance	**0.315**	2.506	0.012
H4c: Gender -> Value and Health Claims Importance	0.020	0.256	0.798
H4d: Age -> Value and Health Claims Importance	−0.002	0.027	0.978
H4e: Education -> Value and Health Claims Importance	**0.149**	2.371	0.018
H4f: Income -> Value and Health Claims Importance	**−0.181**	2.347	0.019
H5a: Objective Pet Food Knowledge -> Natural Ingredients Importance	**0.207**	2.346	0.019
H5b: Subjective Pet Food Knowledge -> Natural Ingredients Importance	**0.481**	6.193	0.000
H5c: Gender -> Natural Ingredients Importance	−0.055	0.936	0.349
H5d: Age -> Natural Ingredients Importance	0.050	0.795	0.427
H5e: Education -> Natural Ingredients Importance	−0.027	0.510	0.610
H5f: Income -> Natural Ingredients Importance	0.005	0.086	0.931
Bold = *p* < 0.05			

## Data Availability

The data presented in this study are available on request from the corresponding author.

## References

[1] Bir C., Widmar N.J.O., Croney C.C. (2017). Stated preferences for dog characteristics and sources of acquisition. Animals.

[2] Bir C., Widmar N.J.O., Croney C.C. (2016). Public Perceptions of Dog Acquisition: Sources, Rationales and Expenditures.

[3] Gates M.C., Walker J., Zito S., Dale A. (2019). Cross-sectional survey of pet ownership, veterinary service utilisation, and pet-related expenditures in New Zealand. N. Z. Vet. J..

[4] German A.J. (2015). Style over substance: What can parenting styles tell us about ownership styles and obesity in companion animals?. Br. J. Nutr..

[5] Volsche S. (2018). Negotiated bonds: The practice of childfree pet parenting. Anthrozoös.

[6] American Pet Product Association 2021–2022 APPA National Pet Owners Survey. https://www.americanpetproducts.org/pubs_survey.asp.

[7] Lundmark F., Berg C., Schmid O., Behdadi D., Röcklinsberg H. (2014). Intentions and values in animal welfare legislation and standards. J. Agric. Environ. Ethics.

[8] Bir C., Croney C.C., Widmar N.J.O. (2019). US Residents’ Perceptions of Dog Welfare Needs and Canine Welfare Information Sources. J. Appl. Anim. Welf. Sci..

[9] Landau R.E., Beck A., Glickman L.T., Litster A., Widmar N.J.O., Moore G.E. (2015). Survey of US veterinary students on communicating with limited English proficient Spanish-speaking pet owners. J. Vet. Med Educ..

[10] Bir C., Widmar N.J.O., Croney C.C. (2016). The Whole “Kitten”-Caboodle: Perceived Differences in Veterinary and General Population Opinions Regarding Cat Behavior and Health. Open J. Vet. Med..

[11] Bir C., Ortez M., Olynk Widmar N.J., Wolf C.A., Hansen C., Ouedraogo F.B. (2020). Familiarity and Use of Veterinary Services by US Resident Dog and Cat Owners. Animals.

[12] Bir C., Wolf C.A., Widmar N.O. (2020). Dog and Cat Owner Demand for Veterinary Service Payment Plans. J. Agric. Resour. Econ..

[13] Lansade L., Trösch M., Parias C., Blanchard A., Gorosurreta E., Calandreau L. (2021). Horses are sensitive to baby talk: Pet-directed speech facilitates communication with humans in a pointing task and during grooming. Anim. Cogn..

[14] Widmar N.O., Bir C., Slipchenko N., Wolf C., Hansen C., Ouedraogo F. (2020). Online procurement of pet supplies and willingness to pay for veterinary telemedicine. Prev. Vet. Med..

[15] Statista (2021). Pet Food Market in the US in 2020. https://www.statista.com/topics/1369/pet-food/.

[16] Rogues J., Csoltova E., Larose-Forges C., Mehinagic E. (2022). Sensory evaluation of pet food products. Nonfood Sesory Practices.

[17] Morelli G., Stefanutti D., Ricci R. (2021). A Survey among Dog and Cat Owners on Pet Food Storage and Preservation in the Households. Animals.

[18] Zicker S.C. (2008). Evaluating pet foods: How confident are you when you recommend a commercial pet food?. Top. Companion Anim. Med..

[19] Vinassa M., Vergnano D., Valle E., Giribaldi M., Nery J., Prola L., Schiavone A. (2020). Profiling Italian cat and dog owners’ perception of pet food quality and their purchasing habits. BMC Vet. Res..

[20] Raditic D.M. (2021). Insights into Commercial Pet Foods. Vet. Clin. Small Anim. Pract..

[21] Xiao Y., Wang H.H., Li J. (2021). A New Market for Pet Food in China: Online Consumer Preferences and Consumption. Chin. Econ..

[22] Montegiove N., Pellegrino R.M., Emiliani C., Pellegrino A., Leonardi L. (2021). An Alternative Approach to Evaluate the Quality of Protein-Based Raw Materials for Dry Pet Food. Animals.

[23] Sanderson S.L. (2021). Pros and Cons of Commercial Pet Foods (Including Grain/Grain Free) for Dogs and Cats. Vet. Clin. Small Anim. Pract..

[24] Corsato Alvarenga I., Dainton A.N., Aldrich C.G. (2021). A review: Nutrition and process attributes of corn in pet foods. Crit. Rev. Food Sci. Nutr..

[25] Park M.E., Um J.B. (2021). Consumer Characteristics in Terms of Pet Food Selection Attributes. J. Agric. Ext. Community Dev..

[26] Bischoff K., Rumbeiha W.K. (2018). Pet food recalls and pet food contaminants in small animals: An update. Vet. Clin. Small Anim. Pract..

[27] Buff P.R., Carter R.A., Bauer J.E., Kersey J.H. (2014). Natural pet food: A review of natural diets and their impact on canine and feline physiology. J. Anim. Sci..

[28] Conlin R., Labban A. (2019). Clustering attitudes and behaviors of high/low involvement grocery shopper. J. Food Prod. Mark..

[29] Montandon A.C., Ogonowski A., Botha E. (2017). Product involvement and the relative importance of health endorsements. J. Food Prod. Mark..

[30] Barone M.J., Norman A.T., Miyazaki A.D. (2007). Consumer response to retailer use of cause-related marketing: Is more fit better?. J. Retail..

[31] Zhang A., Saleme P., Pang B., Durl J., Xu Z. (2020). A systematic review of experimental studies investigating the effect of Cause-Related Marketing on consumer purchase intention. Sustainability.

[32] Kunamaneni S., Jassi S., Hoang D. (2019). Promoting reuse behaviour: Challenges and strategies for repeat purchase, low-involvement products. Sustain. Prod. Consum..

[33] Schifferstein H.N., Desmet P.M. (2010). Hedonic asymmetry in emotional responses to consumer products. Food Qual. Prefer..

[34] Calvo-Porral C., Ruiz-Vega A., Lévy-Mangin J.P. (2018). Does product involvement influence how emotions drive satisfaction?: An approach through the Theory of Hedonic Asymmetry. Eur. Res. Manag. Bus. Econ..

[35] Dotson M.J., Hyatt E.M. (2008). Understanding dog–human companionship. J. Bus. Res..

[36] Durgee J.F. (2008). A commentary on “Understanding Dog–Human Companionship”. J. Bus. Res..

[37] Jyrinki H., Leipamaa-Leskinen H. (2005). Pets as extended self in the context of pet food consumption. Eur. Adv. Consum. Res..

[38] Boya U.O., Dotson M.J., Hyatt E.M. (2015). A comparison of dog food choice criteria across dog owner segments: An exploratory study. Int. J. Consum. Stud..

[39] Trigg J., Thompson K., Smith B., Bennett P. (2016). An animal just like me: The importance of preserving the identities of companion-animal owners in disaster contexts. Soc. Personal. Psychol. Compass.

[40] Knight A., Satchell L. (2021). Vegan versus meat-based pet foods: Owner-reported palatability behaviours and implications for canine and feline welfare. PLoS ONE.

[41] Schleicher M., Cash S.B., Freeman L.M. (2019). Determinants of pet food purchasing decisions. Can. Vet. J..

[42] Kwak M.K., Cha S.S. (2021). A Study on the Selection Attributes Affecting Pet Food Purchase: After COVID-19 Pandemic. Int. J. Food Prop..

[43] Hwang H., Nam S.J. (2021). The influence of consumers’ knowledge on their responses to genetically modified foods. GM Crop. Food.

[44] Raju P.S., Lonial S.C., Mangold W.G. (1995). Differential effects of subjective knowledge, objective knowledge, and usage experience on decision making: An exploratory investigation. J. Consum. Psychol..

[45] Vigar-Ellis D., Pitt L., Caruana A. (2015). Knowledge effects on the exploratory acquisition of wine. Int. J. Wine Bus. Res..

[46] Rihn A., Khachatryan H., Wei X. (2021). Perceived subjective versus objective knowledge: Consumer valuation of genetically modified certification on food producing plants. PLoS ONE.

[47] Park C.W., Mothersbaugh D.L., Feick L. (1994). Consumer knowledge assessment. J. Consum. Res..

[48] Lawley M., Craig J.F., Dean D., Birch D. (2019). The role of seafood sustainability knowledge in seafood purchase decisions. Br. Food J..

[49] Lehberger M., Becker C. (2020). Plant protection practices: How do risk perception, subjective and objective knowledge influence the preference of German consumers. Br. Food J..

[50] Peschel A.O., Grebitus C., Steiner B., Veeman M. (2016). How does consumer knowledge affect environmentally sustainable choices? Evidence from a cross-country latent class analysis of food labels. Appetite.

[51] Grebitus C., Steiner B., Veeman M.M. (2016). Paying for sustainability: A cross-cultural analysis of consumers’ valuations of food and non-food products labeled for carbon and water footprints. J. Behav. Exp. Econ..

[52] Surie M.L. (2014). An exploratory study on the pet food purchasing behaviour of New Zealand consumers. Bachelor’s Thesis (Honors).

[53] Carter R.A., Bauer J.E., Kersey J.H., Buff P.R. (2014). Awareness and evaluation of natural pet food products in the United States. J. Am. Vet. Med Assoc..

[54] Suarez L., Peña C., Carretón E., Juste M.C., Bautista-Castaño I., Montoya-Alonso J.A. (2012). Preferences of owners of overweight dogs when buying commercial pet food. J. Anim. Physiol. Anim. Nutr..

[55] Thompson A. (2008). Ingredients: Where pet food starts. Top. Companion Anim. Med..

[56] Thomas M., Feng Y. (2020). Risk of foodborne illness from pet food: Assessing pet owners′ knowledge, behavior, and risk perception. J. Food Prot..

[57] Zhang J., Wang L., Kannan K. (2019). Polyethylene terephthalate and polycarbonate microplastics in pet food and feces from the United States. Environ. Sci. Technol..

[58] Witaszak N., Waśkiewicz A., Bocianowski J., Stępień Ł. (2020). Contamination of pet food with mycobiota and Fusarium mycotoxins—Focus on dogs and cats. Toxins.

[59] Conway D.M., Saker K.E. (2018). Consumer attitude toward the environmental sustainability of grain-free pet foods. Front. Vet. Sci..

[60] Hobbs L. (2019). Analysis of customer perception of product attributes in pet food: Implications for marketing and product strategy. Master’s Thesis.

[61] Leiva A., Molina A., Redondo-Solano M., Artavia G., Rojas-Bogantes L., Granados-Chinchilla F. (2019). Pet food quality assurance and safety and quality assurance survey within the Costa Rican pet food industry. Animals.

[62] Rombach M., Dean D.L. (2021). Just Love Me, Feed Me, Never Leave Me: Understanding Pet Food Anxiety, Feeding and Shopping Behavior of US Pet Owners in Covidian Times. Animal.

[63] Lemke R.J., Burkholder W.J., Conway C.E., Lando A.M., Valcin S. (2015). An analysis of pet food label usage. J. Consum. Aff..

[64] Lopez A., Vasconi M., Battini M., Mattiello S., Moretti V.M., Bellagamba F. (2020). Intrinsic and Extrinsic Quality Attributes of Fresh and Semi-Hard Goat Cheese from Low- and High-Input Farming Systems. Animals.

[65] Koppel K., Suwonsichon S., Chambers D., Chambers IV E. (2018). Determination of intrinsic appearance properties that drive dry dog food acceptance by pet owners in Thailand. J. Food Prod. Mark..

[66] Pesi U. (2007). Convenience, enjoyment and health: Parallels between marketing human and pet foods. Nutritional Biotechnology in the Feed and Food Industries, Proceedings of Alltech’s 23rd Annual Symposium. The New Energy Crisis: Food, Feed or Fuel? Lexington, KY, USA, 20–23 May 2007.

[67] Anturaniemi J., Barrouin-Melo S.M., Zaldivar-López S., Sinkko H., Hielm-Björkman A. (2019). Owners’ perception of acquiring infections through raw pet food: A comprehensive internet based survey. Vet. Rec..

[68] Morelli G., Bastianello S., Catellani P., Ricci R. (2019). Raw meat-based diets for dogs: Survey of owners’ motivations, attitudes and practices. BMC Vet. Res..

[69] Rigdon E.E. (2016). Choosing PLS path modeling as analytical method in European management research: A realist perspective. Eur. Manag. J..

[70] Hair J.F., Ringle C.M., Sarstedt M. (2011). PLS-SEM: Indeed a silver bullet. J. Mark. Theory Pract..

[71] Sarstedt M., Diamantopoulos A., Salzberger T., Baumgartner P. (2016). Selecting single items to measure doubly concrete constructs: A cautionary tale. J. Bus. Res..

[72] Hair J.F., Hult G.T.M., Ringle C.M., Sarstedt M., Thiele K.O. (2017). Mirror, mirror on the wall: A comparative evaluation of composite-based structural equation modeling methods. J. Acad. Mark. Sci..

[73] Hair J.F., Sarstedt M., Ringle C.M. (2019). Rethinking some of the rethinking of partial least squares. Eur. J. Mark..

[74] Hair J.F., Risher J.J., Sarstedt M., Ringle C.M. (2019). When to use and how to report the results of PLS-SEM. Eur. Bus. Rev..

[75] Henseler J., Ringle C.M., Sarstedt M. (2015). A new criterion for assessing discriminant validity in variance-based structural equation modeling. J. Acad. Mark. Sci..

[76] Hair J.F., Hult G.T.M., Ringle C.M., Sarstedt M. (2017). A Primer on Partial Least Squares Structural Equation Modeling (PLS-SEM).

[77] Fornell C., Larcker D.F. (1981). Evaluating structural equation models with unobservable variables and measurement error. J. Mark. Res..

[78] Han T.I. (2019). Objective knowledge, subjective knowledge, and prior experience of organic cotton apparel. Fash. Text..

[79] Banton S., Baynham A., Pezzali J.G., von Massow M., Shoveller A.K. (2021). Grains on the brain: A survey of dog owner purchasing habits related to grain-free dry dog foods. PLoS ONE.

[80] De Boer J., Boersema J.J., Aiking H. (2009). Consumers′ motivational associations favoring free-range meat or less meat. Ecol. Econ..

[81] Gullo K., Liu P., Zhou L., Fitzsimons G.J., Gneezy A., Griskevicius V., Williams P. (2017). Are my dog’s treats making me fat? The effects of choices made for others on subsequent choices for the self. Advances in Consumer Research.

[82] Vermeer W.M., Bruins B., Steenhuis I.H. (2010). Two pack king size chocolate bars. Can we manage our consumption?. Appetite.

[83] Peura-Kapanen L., Jallinoja P., Kaarakainen M. (2017). Acceptability of convenience food among older people. Sage Open.

[84] Laguna L., Mingioni M., Maitre I., Vanwymelbeke V., Pirttijärvi T., Artigas M.G., Sarkar A. (2016). Perception of difficulties encountered in eating process from European elderlies’ perspective. J. Texture Stud..

[85] Duizer L.M., Robertson T., Han J. (2009). Requirements for packaging from an ageing consumer’s perspective. Packag. Technol. Sci. Int. J..

[86] Park C., Kang C. (2008). Does education induce healthy lifestyle?. J. Health Econ..

[87] Goodman J.K., Paolacci G. (2017). Crowdsourcing consumer research. J. Consum. Res..

